# Initial and cumulative recurrence patterns of glioblastoma after temozolomide-based chemoradiotherapy and salvage treatment: a retrospective cohort study in a single institution

**DOI:** 10.1186/1748-717X-8-97

**Published:** 2013-04-23

**Authors:** Kengo Ogura, Takashi Mizowaki, Yoshiki Arakawa, Masakazu Ogura, Katsuyuki Sakanaka, Susumu Miyamoto, Masahiro Hiraoka

**Affiliations:** 1 Departments of Radiation Oncology and Image-applied Therapy, 54 Kawahara-cho, Shogoin Sakyo-ku, Kyoto 606-8507, Japan; 2 Neurosurgery, Kyoto University Graduate School of Medicine, 54 Kawahara-cho, Kyoto 606-8507, Japan

**Keywords:** Glioblastoma, Recurrence patterns, Temozolomide, Radiotherapy, RANO criteria, Salvage treatment

## Abstract

**Purpose:**

To analyze initial recurrence patterns in patients with newly diagnosed glioblastoma after radiotherapy plus concurrent and adjuvant temozolomide, and to investigate cumulative recurrence patterns after salvage treatment, including surgery, stereotactic radiotherapy, and chemotherapy.

**Methods:**

Twenty-one patients with glioblastoma that recurred after concurrent temozolomide and localized radiotherapy were retrospectively analyzed (11 male, 10 female; median age, 57 years; range, 27–74). Disease progression was assessed by new response criteria proposed by the Response Assessment in Neuro-Oncology Working Group of the American Society of Clinical Oncology. The pattern of recurrence was determined by relationships between locations of recurrent tumors and irradiated doses. Central, in-field, marginal, and out-field recurrences were defined relative to the prescribed isodose line. Distant recurrence was operationally defined as subependymal or disseminated disease. Initial and cumulative patterns of recurrence were evaluated in each patient.

**Results:**

The median follow-up of the recurrent patients was 501 (range, 217–1815) days after initial surgery. Initial recurrences were central in 14 patients (66.7%), in-field in four patients (19.0%), marginal in no patient (0%), out-field in two patients (9.5%), and distant in four patients (19.0%). One patient had both central and in-field recurrences simultaneously, and two had both central and distant recurrences. In the analysis of cumulative recurrence patterns, five patients, who had no scans after initial recurrences, were excluded and the remaining 16 were included. Cumulative recurrences were central in 11 patients (68.8%), in-field in five patients (31.3%), marginal in three patients (18.8%), out-field in five patients (31.3%), and distant in 14 patients (87.5%). Regarding salvage treatments, 11 (52.4%), 11 (52.4%) and 17 (81.0%) patients underwent surgery, stereotactic radiotherapy and chemotherapy, respectively. Eighteen (85.7%) patients had died at the time of analysis, and 16 of them (88.9%) had suffered distant recurrences, which could have been the immediate causes of death.

**Conclusions:**

Recurrence patterns of glioblastoma after radiotherapy plus concomitant and adjuvant temozolomide were mainly central at first, and distant recurrences were often detected during the clinical course. Much better local control and prevention of distant recurrence, including effective salvage treatment, seem to be important.

## Background

Glioblastoma (GBM) is a malignant primary brain tumor with locally aggressive nature and dismal prognosis. The standard therapy for newly diagnosed GBM is maximal safe resection followed by radiotherapy and concurrent/adjuvant chemotherapy with temozolomide (TMZ) [1]. When assessing the response to treatments for GBM, response criteria defined by Macdonald et al. [2] have been widely adopted. The assessment of tumor response is based on the size of the contrast-enhancing tumor on computed tomography (CT) or magnetic resonance imaging (MRI), considering clinical state and corticosteroid dose. Recent studies [3-5], however, have reported a number of limitations to these criteria. The main limitation is that Macdonald’s criteria rely on the change in contrast enhancement. The volume or intensity of contrast enhancement is influenced by various factors. In particular, the emergence of TMZ and antiangiogenic therapy, such as bevacizumab, has made the evaluation of tumor response difficult because of the phenomena of pseudo-progression and pseudo-response.

Recently, recurrence patterns after TMZ-based chemoradiotherapy have been reported [6-10]. These studies were based mainly on Macdonald’s criteria or modified criteria in each institution. New response criteria have been proposed by the Response Assessment in Neuro-Oncology (RANO) Working Group of the American Society of Clinical Oncology (RANO criteria) to overcome the difficulty with Macdonald’s criteria [11]. In this study, we retrospectively analyzed the initial recurrence patterns of GBM after TMZ-based chemoradiotherapy, referring to the RANO criteria, and we also investigated cumulative patterns of failure after salvage treatment, including surgery, stereotactic radiotherapy (SRT), and chemotherapy. To our knowledge, this is the first report of use of the RANO criteria for the retrospective analysis of recurrence patterns of glioblastoma.

## Methods

### Patient population

From March 2006 through February 2011, 47 consecutive patients with newly diagnosed GBM were treated with concurrent TMZ and RT at our hospital. In this study, 10 of those patients were excluded from the analysis for the following reasons: one patient was lost to follow up, two died shortly after the completion of RT, with no follow-up MRI scans available, one had renal failure and could not undergo contrast enhanced MRI scans, two had multifocal lesions and received whole-brain radiotherapy for the initial radiation field, one was part of a clinical study in which target delineation was different from our daily clinical practice, one had another lesion that was suspected to be low-grade glioma on the contralateral side, one underwent hypofractionated SRT as a boost, and one underwent stereotactic radiosurgery as initial treatment at another hospital. This retrospective study is in compliance with the Declaration of Helsinki (Sixth Revision, 2008). All data were collected retrospectively and in accordance with institutional ethical policies.

### Treatment and follow-up

The patients underwent maximal safe tumor resection or stereotactic biopsy and were pathologically diagnosed as GBM. After the diagnosis, they received RT and concurrent/adjuvant TMZ. The dose of TMZ was 75 mg/m^2^/day during the RT and 150-200 mg/m^2^/day for 5 days every 28 days as adjuvant treatment until disease progression. The dosage was reduced or sustained in patients with severe toxicity at the decision of the treating physician.

Patients were evaluated with contrast-enhanced MRI before and after neurosurgery. Then, they were followed up with MRI after the completion of RT and every 1 to 2 months thereafter, or according to clinical symptoms. Patients with suspicious pseudo-progression were observed without changing adjuvant chemotherapy. If the lesions were stable or resolved during follow up, these cases were considered to be pseudo-progression. Positron emission tomography imaging or surgical procedures were performed for differential diagnosis as necessary. When the lesions were continuously growing with or without clinical symptoms, surgical procedures including stereotactic biopsy were recommended for differential diagnosis at the discretion of the treating physician.

### Radiation therapy

**Figure 1:**
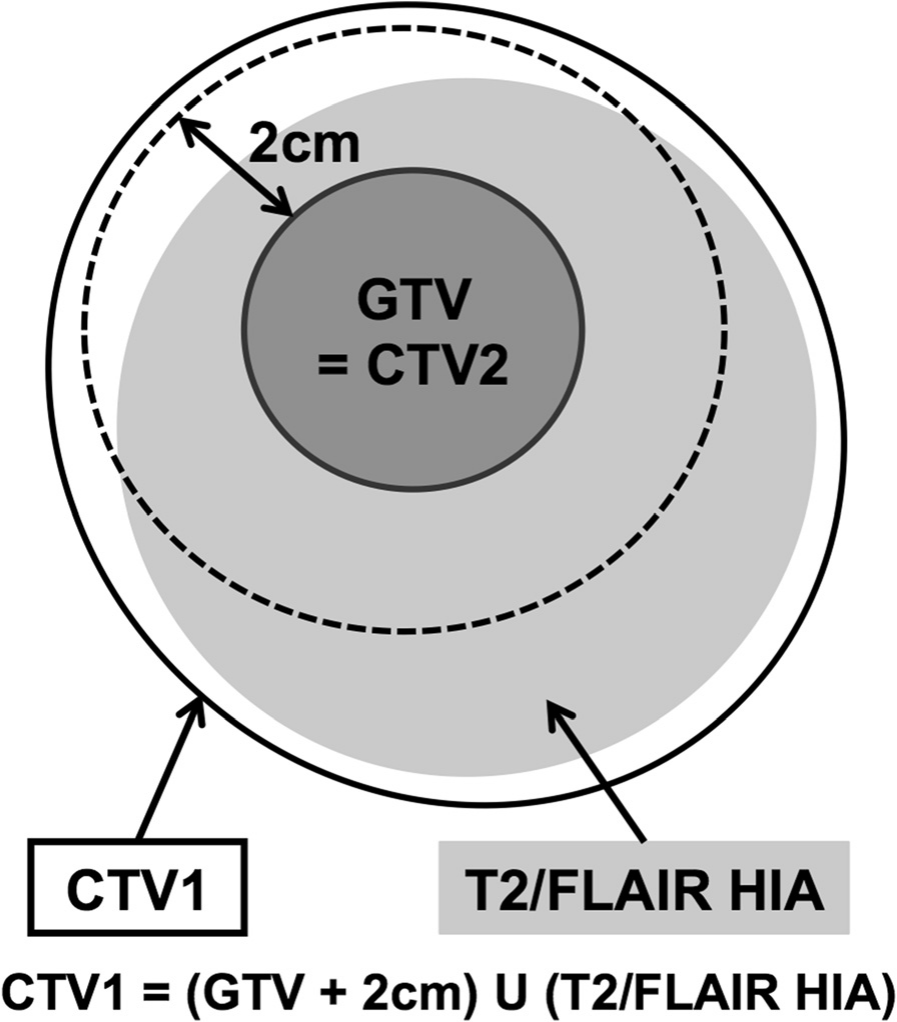
**The target delineation method used in our hospital.** GTV = gross target volume, CTV = clinical target volume, FLAIR = fluid-attenuated inversion recovery, HIA = high intensity area.

Patients were immobilized with thermoplastic masks in the supine position and underwent CT simulation with a 0.25-cm slice thickness. Radiation treatment planning was performed with the Varian Eclipse Treatment Planning System. Preoperative and postoperative MRI images were fused with planning CT images. The gross target volume (GTV) was defined as the sum of the resection cavity and the residual tumor based on postoperative contrast-enhanced MRI. The clinical target volume (CTV) was classified into CTV1 and CTV2. CTV1 was defined by adding a 2-cm margin to the GTV and was modified to include high-intensity areas of T2 or fluid-attenuated inversion recovery (FLAIR) sequences based on pre- and postoperative MRI. CTV2 was identical to the GTV. These are depicted graphically in Figure 1. The CTV1 was manually edited according to the anatomic barriers to tumor spread such as bone, ventricles, cerebral falx, and cerebellar tentorium. The CTV1 was also modified if sparing of radiosensitive organs such as optic nerves, chiasm, and brain stem was needed. Then, the CTV1 and the CTV2 were expanded 0.5 cm to create planning target volume (PTV) in consideration of set-up error and patient motion, named as PTV1 and PTV2, respectively.

Of the 37 eligible patients, 32 (86.5%) were treated with three-dimensional conformal radiotherapy (3D-CRT). The dosage was 50 Gy in 25 fractions for PTV1 followed by 10 Gy in five fractions for PTV2. The remaining five patients (13.5%) were treated with intensity modulated radiation therapy (IMRT) using a simultaneous integrated boost (SIB) technique. The dose prescription was as follows: 54 Gy in 30 fractions (1.8 Gy per fraction) was prescribed for PTV1, and 60 Gy for PTV2 in 30 fractions (2 Gy per fraction) simultaneously.

### Analysis of recurrences

We retrospectively analyzed the response to treatment and the patterns of recurrence, referring to the RANO criteria [11]. Briefly, progression is defined as: increase in 25% of the product of perpendicular diameters of enhancing lesions, a significant increase in the T2/FLAIR non-enhancing component, appearance of new lesions, and clinical deterioration not attributable to causes other than the tumor or reduction in corticosteroid dose. Within 12 weeks after completion of progressive disease can be defined only if there is a new lesion(s) outside of the radiation field or if there is unequivocal evidence of a viable tumor on histopathological sampling. According to these criteria, we took into account both pseudo-progression and pseudo-response. We assessed tumor progression by not only the contrast-enhancing component but also the non-enhancing one for patients treated with antiangiogenic therapy. Corticosteroid dose and clinical status were also considered.

When tumors were diagnosed as progressive, the MRI scans at that time were registered with the initial planning CT using the Varian Eclipse software (ver. 8.6). Then, the relationship between the location of recurrent tumors and the delivered radiation doses was assessed. The recurrent tumors were delineated as recurrent tumor volume (RTV), and the dose distribution of initial radiotherapy was overlaid. The patterns of recurrence were categorized into five groups: central if more than 95% of the RTV was included in the 95% isodose line of 60 Gy, in-field if more than 95% of the RTV overlapped with the 95% isodose line of 50 Gy (for 3D-CRT; 54 Gy for IMRT), marginal if 95% or less of the RTV overlapped, out-field if none of the RTV overlapped, and distant when subependymal or disseminated recurrence was detected on MRI scans or cerebrospinal examination. Initial and cumulative recurrence patterns were estimated according to these definitions. In the assessment of cumulative recurrence, each pattern of recurrence was counted every time a new recurrent lesion was detected in follow-up MRI scans after initial recurrence. Patients who had no successive scans after initial recurrence were excluded from the analysis of cumulative recurrence patterns.

In our hospital, antiangiogenic therapy was not used as a first regimen, and we usually did not need to consider non-enhancing lesions when analyzing initial patterns of recurrence. On the other hand, we should consider those lesions when analyzing cumulative recurrence because some patients were treated with antiangiogenic agents as salvage therapy. For recurrent tumors with no enhancement, T2/FLAIR images were registered with the initial planning CT, and RTV was delineated as continuously growing T2/FLAIR high intensity signal on stable or increasing doses of corticosteroids.

Overall survival (OS) and progression-free survival (PFS) of recurrent patients were estimated. OS was calculated from the date of initial surgery to that of death or last follow-up. PFS was calculated from the date of initial surgery to that of disease progression defined by the RANO criteria. Both were measured using the Kaplan-Meier method.

## Results

### Survival

**Figure 2:**
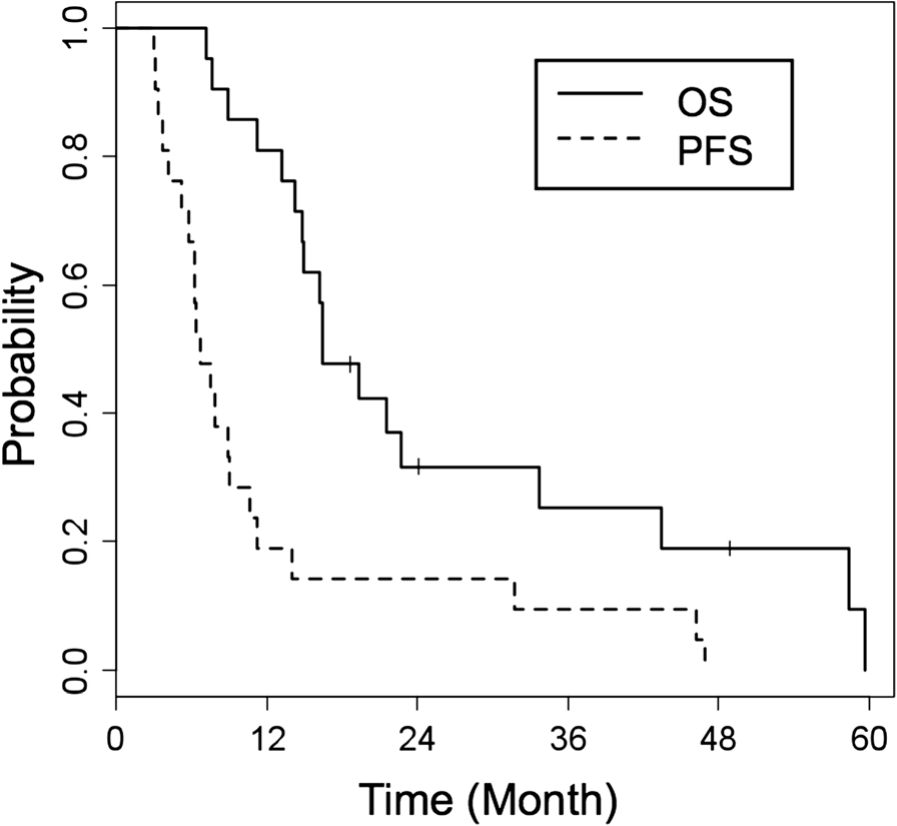
**Overall survival and progression-free survival.** Overall survival (OS) and progression-free survival (PFS) of 21 recurrent patients were estimated using the Kaplan-Meier method.

To date, of 37 eligible patients, 21 (56.8%) have been diagnosed with progressive disease. The characteristics of recurrent patients are summarized in Table 1. Eighteen (85.7%) died at a median follow-up of 501 (range, 217–1815) days after initial surgery. The 1-year and 2-year OSs of recurrent patients were 81.0% (95% confidence interval [CI], 65.8–99.6) and 31.7% (95% CI, 16.7–60.4), respectively. The 6-month and 12-month PFSs of recurrent patients were 66.7% (95% CI, 49.2–90.2) and 19.1% (95% CI, 7.9–46.0), respectively (Figure 2).

**Table 1:** Characteristics of 21 recurrent patients

**Characteristics**	**No. (%)**
Gender	
Male	11 (52.4)
Female	10 (47.6)
Age (years)	
Median	57
Range	27-74
Karnofsky performance status	
< 70	6 (28.6)
70–80	11 (52.4)
90–100	4 (19.0)
RTOG-RPA classification	
III	3 (14.3)
IV	7 (33.3)
V	7 (33.3)
VI	4 (19.0)
Extent of surgery	
Gross total resection	8 (38.1)
Subtotal resection	3 (14.3)
Partial resection	8 (38.1)
Biopsy	2 (9.5)

RTOG-RPA: Radiation Therapy Oncology Group recursive partitioning analysis.

### Patterns of failure

**Figure 3:**
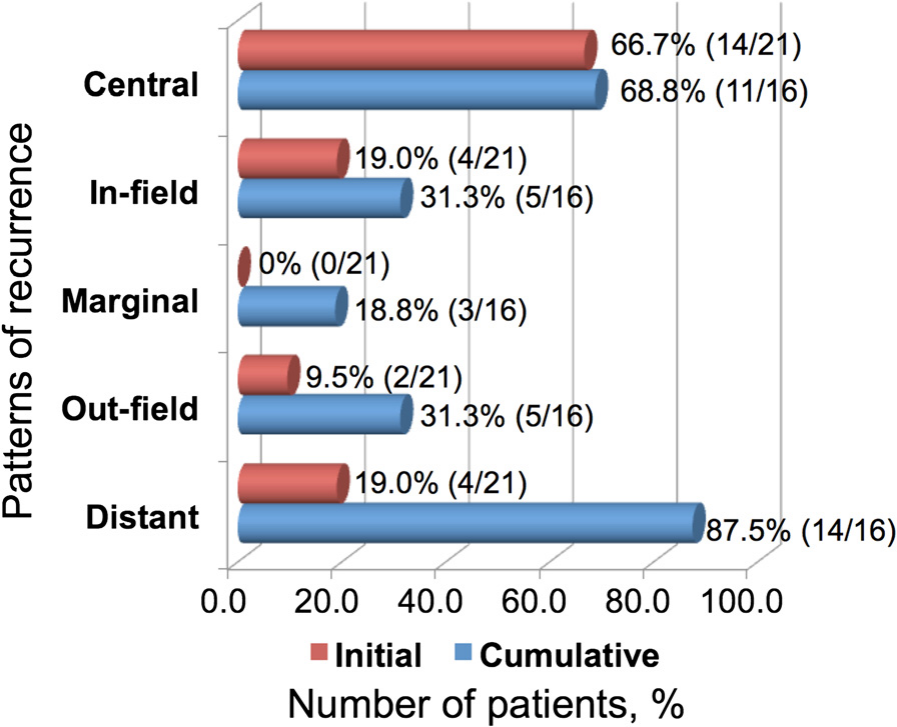
**Summary of initial and cumulative recurrence patterns.** Initial and cumulative recurrence patterns in 21 and 16 recurrent patients, respectively. The red bars indicate initial recurrences and the blue bars cumulative recurrences.

**Figure 4:**
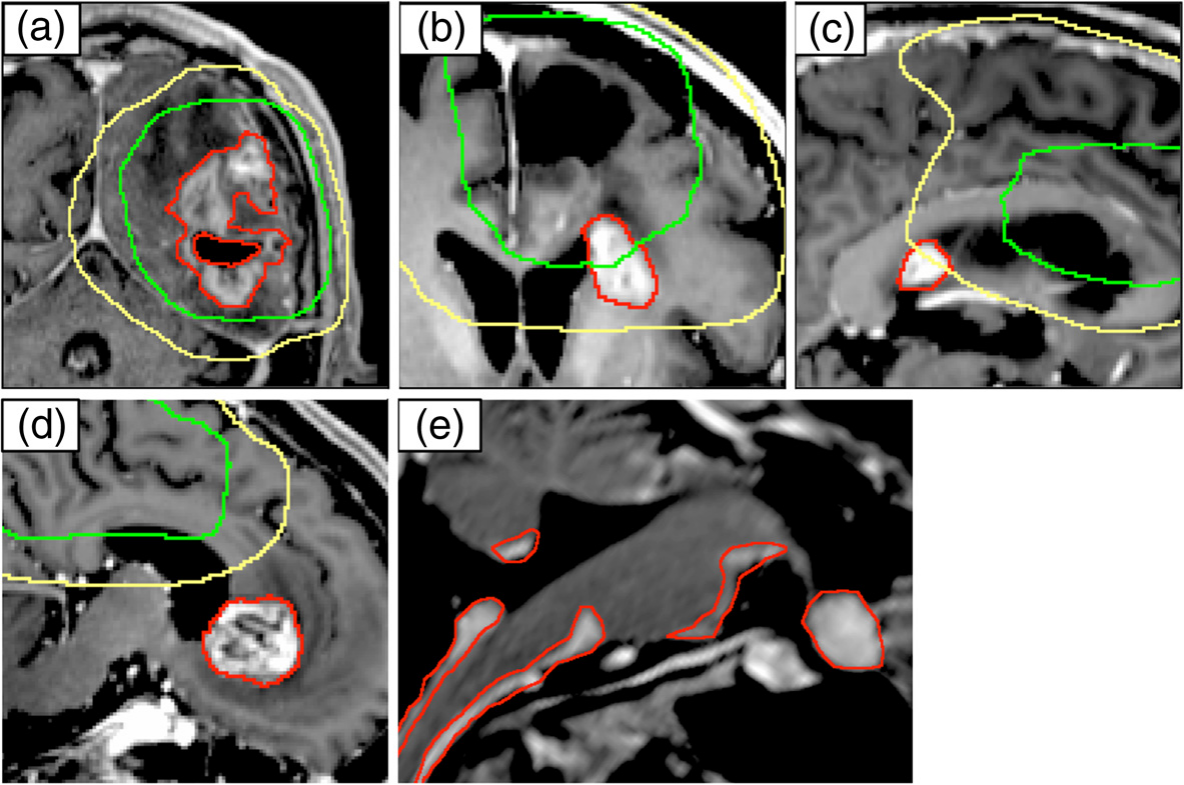
**Examples of recurrence patterns.** Examples of recurrence patterns on T1-weighted magnetic resonance imaging with contrast: central (**a**), in-field (**b**), marginal (**c**), out-field (**d**), and distant recurrences (**e**). Red contours indicate recurrent tumors. Green and yellow lines indicate 95% isodose lines of 60 Gy and 50 Gy, respectively.

The results of recurrence patterns and typical cases are shown in Figures 3 and 4, respectively. Details of recurrence patterns and salvage treatments are summarized in Table 2. All of the initial recurrences were diagnosed as new or enlarged contrast-enhancing lesions on MRI scans. Central recurrence was most often observed as the initial recurrence. One patient had both central and in-field recurrences simultaneously, and two had both central and distant recurrences. The median times from initial surgery to initial failure were 191 (range, 90-1430) days for central/in-field recurrences and 161.5 (range, 111–425) days for other recurrences. Regarding tumor locations, 14 of 21 recurrent patients had primary tumors adjacent to the ventricles and four of them (28.6%) had distant recurrences as initial patterns; two had central and distant recurrences simultaneously, and the other two had only distant recurrences. On the other hand, none of the remaining seven patients with primary tumors not adjacent to the ventricles had distant recurrences as initial patterns. Regarding cumulative recurrences, 16 patients had successive follow-up MRI scans after initial recurrence and were included in the analysis. The remaining five patients underwent no follow-up imaging after initial recurrence because of poor performance status due to progressive disease in four patients (all had distant recurrences) and changing hospital in the other patient. Central and distant recurrences were the most often detected (Figure 3, Table 2). Distant recurrence was observed in almost all the patients who died (16 of 18 patients); 14 of these 16 patients had an uncontrollable local lesion(s) before distant recurrence occurred. The median survival time after the diagnosis of distant recurrence was 149 (range, 63–394) days.

**Table 2:** Details of recurrence patterns and salvage treatments

**Patient no.**	**Patterns of recurrence**						**Salvage treatment**^**b**^			**Current status**
	**Initial**	**Cumulative**^**a**^								
		**Central**	**In-field**	**Marginal**	**Out-field**	**Distant**	**Surgery**	**SRT**	**Chemo**	
1	Distant	-	-	-	-	-	0	0	-	Dead
2	Central	3	0	1	0	2	Central (1)	Central(1)	ICE, BEV	Dead
								Marginal (1)		
3	Distant	-	-	-	-	-	0	0	ICE	Dead
4	Out-field	0	0	0	2	0	0	Out-field (2)	ICE, BEV, Others	Dead
5	Central	1	0	0	0	1	Central (2)	Central (1)	ICE	Dead
6	Central	1	0	0	0	0	Central (2)	0	ICE	Dead
7	Central	2	0	1	0	2	Central (2)	Central (1)	ICE, BEV, Others	Dead
8	Central	2	0	0	0	1	0	0	ICE, BEV	Dead
9	In-field	1	0	1	2	1	Central + In-field (1)	Marginal (1)	ICE, BEV	Dead
10	Central	0	1	0	1	1	Central (1)	0	ICE, BEV	Dead
11	Central	2	1	0	0	1	Central (1)	Central (2)	ICE, BEV, Others	Dead
								In-field (1)		
12	Out-field	0	0	0	2	1	0	Out-field (1)	ICE, BEV	Alive
13	Central	0	0	0	0	1	0	0	ICE	Dead
14	Central	2	0	0	0	1	Central (2)	Central (1)	ICE, BEV	Dead
15	In-field	-	-	-	-	-	0	In-field (1)	-	Alive
16	Central, In-field	1	1	0	0	1	Central (1)	Central +	ICE, BEV	Dead
								In-field (1)		
17	Central, Distant	-	-	-	-	-	0	0	-	Dead
18	Central	1	1	0	0	1	Central (1)	0	ICE	Dead
							In-field (1)			
19	Central	1	0	0	0	1	Central (2)	0	ICE	Dead
20	In-field	0	1	0	1	1	0	In-field (1)	ICE, Others	Alive
								In-field + Out-field + Distant (1)		
21	Central, Distant	-	-	-	-	-	0	0	-	Dead

BEV: bevacizumab.

ICE: ifosfamide, carboplatin, and etoposide.

SRT: stereotactic radiotherapy.

^a^Cumulative recurrence patterns were classified and counted each time a new recurrent lesion was detected in follow-up MRI scans after the initial recurrence. Five patients had no scans after the initial recurrence and were excluded from the analysis of cumulative recurrence.

^b^Regarding local salvage, recurrence patterns of treated lesions are described; repeat local salvage numbers are also indicated.

Eleven patients (52.4%) underwent salvage surgery for recurrence once or twice; all had central lesions, and two had in-field lesions. The time between initial surgery and first salvage surgery was a median of 262 (range, 92–1470) days. Eleven patients (52.4%) underwent salvage SRT for recurrence once or more; six had central lesions, four had in-field lesions, two had marginal lesions, three had out-field lesions, and one had a subependymal lesion. The median time between initial surgery and first SRT was 392 (range, 152–1498) days. Fifteen patients (71.4%) received surgery and SRT together as local salvage. At the time of initial recurrence, 10 patients underwent local salvage for central lesions, but nine of these suffered from central recurrence again. Local salvage was performed once in three patients, twice in seven patients, three times in four patients, and four times in one patient. Twelve of these 15 patients had died at the time of analysis, and 10 of the 12 patients had subependymal or disseminated disease at the last follow-up MRI.

Patients underwent a median of four cycles of adjuvant TMZ chemotherapy before tumor progression (range, 0–44 cycles). Seventeen patients (81.0%) underwent salvage chemotherapy after the initial recurrence. Seventeen patients (81.0%) received ifosfamide, carboplatin, and etoposide (ICE) as a first salvage chemotherapy regimen. Other regimens were as follows: bevacizumab in nine patients (42.9%), an alternative regimen of TMZ in two patients, nimustin (ACNU) and TMZ in one patient, irinotecan and TS-1 in one patient, and etoposide by oral administration in one patient. With regard to pseudo-response, three of the nine patients (33.3%) had continuously growing tumors with no contrast enhancement during treatment with bevacizumab and were diagnosed with progressive disease. After the treatment with bevacizumab, first recurrence patterns were local in five patients, distant in three patients, and both in one patient.

Pseudo-progression was observed in two of 21 recurrent patients (9.5%), 20 and 57 days after the completion of initial RT. On the other hand, it was observed in six of 16 non-recurrent patients (37.5%) at a median of 73 (range, 34–260) days after the completion of initial RT, and these patients have shown no clear evidence of progressive disease to date. Pseudo-progression was diagnosed by surgical/histological assessment in five patients and by follow-up MRI scans in the remaining three patients. From a slightly different viewpoint, nine of 37 patients (24.3%) were suspected to have progression or pseudo-progression within 12 weeks after the completion of initial RT. The proportion of pseudo-progression was accordingly 55.6% (five of nine contrast-enhancing lesions) within 12 weeks.

## Discussion

In our study, central recurrence was main pattern of initial recurrence, and this finding was consistent with other recent reports [6-10]. In the era of TMZ, the initial pattern of failure still seems to be central and seems to be unchangeable. A comparison between our study and previously reported studies is summarized in Table 3. Target delineation and method of analyzing the pattern of recurrence were slightly or sometimes quite different at each institution. In our hospital, the target delineation was relatively small in both initial and boost fields. The pattern of recurrence showed, however, almost the same tendency. The proportion of central recurrence seemed to be slightly lower in our study. This would be due to the method of analysis. Our clinical target volume in the boost field was smallest, and therefore the volume of recurrent tumors would easily protrude outside the 95% isodose line of 60 Gy due to the nature of the analysis. The proportion of central and in-field recurrences taken together was 81.0% (17 of 21 patients) in our study. This value was almost the same as in previous reports. Additionally, the proportion of marginal recurrence was fairly low in our hospital. These results suggest that a larger field seems to be unnecessary, which can help limit the irradiated volume of normal brain tissues.

**Table 3:** Summary of studies of recurrence patterns of glioblastoma in the temozolomide era

**Author**	**Clinical target delineation and prescribed dose**		**Analyzed recurrent patients**	**Proportion of central recurrence**
**(year)**	**CTV1 (initial field)**	**CTV2 (boost field)**		
Brandes et al. [6]	Enhanced tumor area according to preoperative imaging plus 2–3 cm	None (identical with initial field)	79	72.2%^a^
	60 Gy in 30 fractions			
Milano et al. [7]	Edema plus 2 cm	Residual tumor/resection cavity plus 2–2.5 cm	39	80%^b^
	46–50 Gy in 23–25 fractions			
		60 Gy in 30 fractions		
Minniti et al. [8]	Residual tumor/resection cavity plus 2 cm	Residual tumor/resection cavity plus 1–2 cm	105	75.2%^c^
	50 Gy in 25 fractions	60 Gy in 30 fractions		
McDonald et al. [9]	Edema plus 0.5–1.2 cm	Residual tumor/resection cavity plus 0–1 cm	41	78%^d^
	46–54 Gy in 23–30 fractions			
		60 Gy in 30 fractions		
Dobelbower et al. [10]	Primary tumor and surrounding edema plus 0.5 cm on postoperative imaging	Residual tumor/resection cavity plus 0.5 cm	20	90%^a^
		60Gy in 30 fractions		
	46 Gy in 23 fractions			
Present Study	Residual tumor/resection cavity plus 2 cm and edema	Residual tumor/resection cavity plus 0 cm	21	66.7%
	50–54 Gy in 25–30 fractions	60 Gy in 30 fractions		

CTV: clinical target volume.

^a^The term “central” was not actually used in the report. Instead, “in-field” recurrence was defined as 80% of the tumor recurrence residing within the prescribed 95% isodose surface.

^b^Central recurrence was defined as growth of the original tumor or arising tumor(s) from the resection cavity.

^c^Central recurrence was defined as more than 95% of the recurrence volume within the 95% isodose line of 60 Gy.

^d^Central recurrence was defined as more than 95% of the recurrence volume inside the 100% isodose line of 60 Gy.

No optimal treatment volume for glioblastoma has yet been established [12]. For example, the guidelines for target delineation of the European Organization for Research and Treatment of Cancer (EORTC) and the Radiation Therapy Oncology Group (RTOG) are quite different. In the EORTC guidelines, the CTV is defined as the contrast-enhancing lesion plus a 2–3 cm margin and is not changed between the initial and boost radiation field [1]. On the other hand, in the RTOG guidelines, the CTV is defined as peritumoral edema plus 2 cm in the initial field and the residual tumor plus 2 cm in the boost field (e.g., RTOG 0525 and RTOG 8525 trials) [8]. In our hospital, the treatment volume is relatively small, especially in the boost field. Since the 1980s, the pre-conformal radiotherapy era, initial and boost field techniques have been used continuously. The distances from the tumor to the field edge were 3 and 1 cm in the initial and boost fields, respectively. Next, the margin from the tumor to the field edge has been consistent since the 1980s to-date. In the pre-TMZ era, Shibamoto et al. [13,14] reported that initial relapse developed within the irradiated volume in almost 90% GBM patients and marginal recurrence was rare in our hospital. Additionally, Chang and colleagues [15] at the University of Texas M.D. Anderson Cancer Center (MDACC) reported initial recurrence patterns with a limited radiation field. In the boost field, target delineation of CTV at MDACC was the same as in our hospital. In the initial field, the CTV was GTV plus 2 cm without intentional inclusion of peritumoral edema, which was smaller than in our hospital. Nevertheless, almost all initial recurrences developed within the full-dose volume. Considering these findings, we think smaller target volumes may be regarded as valid. The efficacy using smaller target volumes needs to be demonstrated in future prospective studies.

For recurrent GBM, we actively perform salvage therapy including surgery and SRT. The overall survival in our study seemed to be fairly good even though it was estimated with only recurrent patients (Figure 2). We believe that this prolonged survival was attributable to active local salvage therapy, and salvage chemotherapy with ICE treatment, which is our first regimen for recurrent GBM [16]. On the other hand, local recurrences were continuously observed after each salvage therapy, and distant recurrences became apparent in almost all the patients. Milano et al. [7] reported cumulative recurrence patterns and showed that distant recurrences were often seen for those surviving longer. This could be interpreted to suggest that patients may survive longer until distant recurrence occurs, whereas it is not fully clarified whether better local control including salvage therapy contributes to longer survival. In our results, almost all the patients who died with distant recurrence suffered from continuous local failures until distant recurrence occurred. Once the distant recurrence was apparent, the prognosis was very poor, although it was difficult to know whether the direct cause of death was local and/or distant failure. We would like to propose at least two hypotheses: first, distant recurrence occurs due to continuous failure of local control; second, distant recurrence is the manifestation of disease that is not detectable at the time of initial treatment. To our knowledge, no useful approaches exist after distant recurrence. A strategy to prevent distant recurrence, including much better local control and salvage treatment, seems to be important.

On the other hand, genetic background also seems to influence recurrence patterns. Brandes et al. [6] showed that the patterns of recurrence are correlated with O^6^-methylguanine-DNA methyltransferase (MGMT)-promoter methylation status. They reported that patients with MGMT unmethylated status were more likely to have central recurrences than were those with MGMT methylated status. In this report, we did not survey the status of MGMT methylation and could not conclude how this status influenced on our results. Treatment strategy according to biological information such as genetic status seems to be preferable in the future.

The presentation of pseudo-progression has been reported to occur at 1–10 months and commonly within 3 months after the completion of initial chemoradiotherapy with TMZ [17]. The precise mechanism of pseudo-progression has not been fully elucidated, but it has been suggested that this phenomenon represents a continuum between the subacute radiation reaction and treatment-related necrosis [17-19]. A recent study [20] revealed that this phenomenon can be predicted by MGMT-promoter methylation status and could be a potential marker of survival benefit after TMZ-based chemoradiotherapy for GBM. In our study, although we did not assess MGMT status, pseudo-progression was observed more often in non-recurrent patients (37.5%; six of 16 patients) than recurrent patients (9.5%; two of 21 patients). This result was consistent with the previous reports and suggests that pseudo-progression could be a predictive marker of better clinical outcome.

Limitations of our study were its retrospective nature, the heterogeneity of salvage treatment, the absence of information on genetic background, and the small sample size. However, this study provides some evidence based on new response criteria and showed the same tendency of recurrence pattern as previous reports. To our knowledge, this is the first report of use of the RANO criteria for the retrospective analysis of both initial and cumulative recurrence patterns of glioblastoma. Furthermore, MRI was conducted relatively frequently in our institution, which helped us more precisely know the change in tumor regression or progression and the cumulative recurrences. Additionally, the target delineation with limited margin was thought to support other recent reports and to be preferable in future treatment, although only a prospective study with survival as an endpoint can resolve this.

## Conclusions

The initial recurrence pattern of GBM was mainly central, but scrupulous attention to pseudo-progression is necessary during early periods after initial treatment. After initial recurrence, distant recurrence was also frequently observed. Much better local control and prevention of distant recurrence, including effective local salvage treatment, seem to be important.

### Consent

Written informed consent was obtained from the patient for publication of this report and any accompanying images.

## Abbreviations

3D-CRT: Three-dimensional conformal radiotherapy; BEV: Bevacizumab; CI: Confidence interval; CT: Computed tomography; CTV: Clinical target volume; EORTC: European Organization for Research and Treatment of Cancer; FLAIR: Fluid-attenuated inversion recovery; GBM: Glioblastoma; GTV: Gross target volume; ICE: Ifosfamide, carboplatin, and etoposide; IMRT: Intensity-modulated radiation therapy; MDACC: M.D. Anderson Cancer Center; MGMT: O^6^-methylguanine-DNA methyltransferase; MRI: Magnetic resonance imaging; OS: Overall survival; PFS: Progression-free survival; PTV: Planning target volume; RANO: Response assessment in neuro-oncology; RTOG: Radiation Therapy Oncology Group; RTV: Recurrent tumor volume; SIB: Simultaneous integrated boost; SRT: Stereotactic radiotherapy; TMZ: Temozolomide.

## Competing interests

The authors declare that they have no competing interest.

## Authors’ contributions

KO and TM conceived the study, and participated in its design and coordination. All authors participated in the acquisition/analysis of the data. KO and TM drafted the manuscript. YA, MO, KS, SM, and MH critically reviewed/revised the manuscript. All authors read and approved the final manuscript.
